# Interactions among factors affecting stillbirths in Egyptian buffaloes (Bubalus bubalis)

**DOI:** 10.1007/s11250-025-04402-x

**Published:** 2025-04-24

**Authors:** Ali Ali El-Raghi, Walaa M. Essawi, Mahmoud A. E. Hassan, Nesrein M. Hashem, Sameh A. Abdelnour

**Affiliations:** 1 Department of Animal, Poultry, and Fish Production, Faculty of Agriculture, Damietta University, Damietta, 34517 Egypt; 2 Department of Theriogenology, Faculty of Veterinary Medicine, Aswan University, Aswan, 81528 Egypt; 3 Agriculture Research Center, Animal Production Research Institute (APRI), Ministry of Agriculture, Dokki, 12619 Giza Egypt; 4 Department of Animal and Fish Production, Faculty of Agriculture, Alexandria University, Alexandria, 21545 Egypt; 5 Animal Production Department, Faculty of Agriculture, Zagazig University, Zagazig, 44511 Egypt

**Keywords:** Egyptian buffaloes, Immunity, Metabolic changes, Risk factors, Stillbirth

## Abstract

In buffaloes, stillbirth (SB) is a major source of reproductive and economic losses. Hence, the objectives of this study were: 1) investigating the relationship between potential risk factors (body condition score [BCS], gestation period, calving season, calf sex, and dam parity) and SB occurrence in Egyptian buffaloes; and 2) identifying blood metabolites, the redox status, and immune-inflammatory attributes in calves that may be related to SB. The incidence of SB was 6.64%. Among the evaluated risk factors, BCS was a significant risk factor for SB. There was a 73.7% lower odds (lower odds odd ratio, OR = 0.246) of SB for dams with a gestation length ≥ 305 days, compared to those with a gestation length < 305 days. The risk of SB decreased steadily with increasing dam parity. The odds of SB were 2.48 times higher in male calves compared to female calves. In comparison to the spring season, the probability of SB doubled during the summer season. Calf blood serum analysis showed that SB-born calves had higher blood biochemical and cytokines alterations than normal-born calves. On the other hand, immunoglobulins and glutathione peroxidase were significantly lower in SB-born calves. Our results indicated that factors related to the induction of inflammation and/or disrupted immune system responses, such as obesity, high temperature, and oxidative stress, are the main evoking factors for SB in buffaloes; therefore, protective measures against SB in Egyptian buffaloes should be based on controlling these factors, either by nutritional interventions or management practices.

## Introduction

The buffalo (Bubalus bubalis) holds great promise for animal production in many countries, contributing to meat and/or milk production, particularly in tropical and subtropical regions (Zhang et al. 2020). However, buffaloes are characterized as a poor breeder in general because of late maturity, seasonal breeding, long postpartum anestrous and calving interval, poor expression of estrus and sub-optimal conception rate (El-Bayomi et al. 2018),which may further be complicated by stillbirth (SB). Clinical observation has shown that the stillbirth is one of the major undesirable calving – related disorders in buffaloes, accounting for approximately 42% of all reproductive problems; (SB) is a frequently repeated reproductive disorder in buffalo heifers (Ilieva and Peeva 2008). SB leads to the death of the neonate (calf) shortly before, during, or shortly after parturition (24 – 48h) following a full-term pregnancy (Berglund et al. 2003; Mee et al. 2013). In addition to the negative effects of SB on animal welfare, SB results in considerable economic losses due to the loss of the value of the calves available for sale and replacement (Little and Kay 1979; Meyer et al. 2001; Mahnani et al. 2018), dam mortality, decreased fertility, premature the culling, and redundant veterinary service costs around calving (Meijering 1984; Chassagne et al. 1999; Ilieva and Peeva 2008; Mahnani et al. 2018).

SB can be related to either non-infectious (dystocia) or infectious reasons (Mee and Szenci 2012; Mee et al. 2013). Moreover, other than diseases, several studies have shown that the major risk factors for SB comprise age at first calving (Chassagne et al. 1999), breed of dam, calving management, feto-maternal health state, breeding system, gestation feeding and length (López Helguera et al. 2016), sire, season, and calf sex (Mee et al. 2013). Despite knowledge of the many risk factors for SB, there are many idiopathic reasons for SB; specifically, SB may occur during eutocia (calves seem clinically normal with no clear causes for calves death; Berglund et al. 2003).

The incidence of SB is also believed to be related to environmental and/or physiological factors that can trigger inflammatory responses, oxidative stress, and other metabolic disorders, either in dams or fetuses; these areas have gained attention in an attempt to understand the interplay between these factors and the occurrence of SB (Gavilanes et al. 2009; Jawor et al. 2018). In fact, oxidative stress and impaired immune-inflammatory responses may contribute to placental dysfunction and fetal growth restriction and development, increasing the risk for SB (Baker et al. 2021). In this context, Jawor et al. (2018) noted differences in the perinatal immuno/inflammatory responses of live-born and stillborn calves with or without utero-infections. Thus, investigating changes in the blood metabolites, redox status, and immune-inflammatory indicators of newborn calves may provide important clues for SB-driven reasons by reflecting the major disorders that occur around birth and lead to mortality. To date, little is known about the relationship between these biological biomarkers and the incidence rate of SB in buffalo calves. Identifying the possible interplay between biological biomarkers and SB may offer insight into unexplained mortality and predict SB, even if the cause of death cannot be diagnosed. Accordingly, the aim of the present study was to investigate the risk factors associated with SB in Egyptian buffaloes and to evaluate the serum metabolic profile, redox status, and immuno-inflammatory-related biomarkers in normal-born and SB-born calves.

## Materials and methods

### Animals ethical

An animal, handling and biological samples collection was approved by the Zagazig University—Institutional animal care and Use committee, ZU-IACUC, with approval number: ZU-IACUC/2/F/366/2022.

### Records and animal management

Data encompassing calf and dam identification numbers, body condition score (BCS) of the dam evaluated using a 5-point scale (Wildman et al. 1982; Anitha et al. 2011) as well as calving year, season, gestation period, calf sex, parity of the dam, and the occurrence of SB (recorded as calf dead at birth or within 24 h after parturition) were collected from 1,249 records over three consecutive years from 2020 to 2023. The records were obtained from 364 Egyptian buffaloes (Bubalus bubalis), belonging to three private farms (El-Shorouk, El-Taaween, and El-Omda) located in Al Sharkia governorate, Egypt. All three farms were medium-scale farms and followed the same management and breeding systems applied on most buffalo farms in Egypt (Abdel-Salam and Fahim 2018).

Buffaloes were housed in the same shed, where 50% of the yard area was sheltered, and the animals had free access to open air. All buffaloes were clinically healthy, aged between 3 and 15 years and weight of 350–550 kg. The average milk yield was 8.0 ± 2.0 kg/day. Animals were milked twice daily (at 6 a.m. and 6 p.m.) by hand. The insemination system used on all three farms was natural mating by gathering fertile sires with females for a period of two consecutive estrous cycles. Animals fed on forage dry matter (Trifolium alexandrinum, Egyptian clover, and/or alfalfa) together with a concentrate mixture (1.5 kg per animal for body maintenance, and an extra 0.5 kg concentrate/buffalo fed 2–3 weeks before the expected calving date) with ad libitum access to water. The concentrate mixture was composed of 30% barley, 21% yellow corn, 20% soybean meal, 25% wheat bran, 2% dicalcium phosphate, 1% sodium chloride, and 1% premix. Wheat straw was available ad libitum. These rations provided 12% crude protein and 67% total digestible nutrient (Paul et al. 2002; Paul and Lal 2010).

For health management, buffaloes were vaccinated for blackleg, foot and mouth disease, and anthrax. Young calves were vaccinated using Brucella abortus strain 19 and more recently with Rb51. Additionally, herds were tested for brucellosis and tuberculosis on a quarterly basis.

### Calves and blood sampling

At the last year of the study, 2023, a total of 10 stillborn calves with normal appearance and idiopathic death reasons, born after full-term pregnancies (≥ 310 days) and without signs of dystocia, were subjected to blood sampling at or just after parturition (Jawor et al. 2018). The case inclusion criteria were full – term (≥ 310 d of gestation), died within 24h of birth. Calves which born dead were excluded from the study. Blood sampling to death interval was, on average 5.3 ± 1.3 h, and varied between 4 to 7 h. Blood samples were collected from the jugular vein using syringes and immediately divided into clean sterile tubes. For the control group, 10 normal-born calves (born after a normal pregnancy length, ≥ 310 days), were subjected to blood sample collection from the jugular vein. None of the calves in either group received colostrum until the time of blood sampling. Each blood sample was centrifuged at 3,000 rounds per minute (rpm) for 15 min. The serum was separated and kept at − 20 °C until the time of analysis.

### Blood biochemical attributes

In order to evaluate metabolic status and liver, kidney, and pancreas functions of live-born and stillborn calves, serum samples were analyzed for total proteins, cholesterol, AST (aspartate) and ALT (alanine) aminotransferases, bilirubin, GGT (gamma-glutamyl transferase), and LDH (lactate dehydrogenase), uric acid, creatinine, amylase, and lipase using an automated analyzer (Olympus AU2700 system reagent, Olympus Diagnostica GmbH, Ireland). The analytical performances of analyzer and the reagents provided by Olympus were evaluated according to the French Society of clinical Biology guidelines and have been found accurate and easy to use (Lasnier et al. 2000).

### Redox status attributes

Redox status indicators of blood serum, including TAC (total antioxidant capacity), SOD (superoxide dismutase), MDA (malondialdehyde), GPx (glutathione peroxidase were evaluated spectrophotometrically procedure (Uh 5300 Hitachi spectrophotometer, Tokyo, Japan) and employed commercial kits acquired from Bio diagnostic, 29 Tahreer St, Dokki, Giza, Egypt. The linearity of analyze was up to 2 mM/l for TAC (CAT. NO. TA 25 13) and up to 100 nmol/ml for MDA (CAT. No. MD 25 29). Activities of antioxidant enzymes including SOD (CAT. No. SD 25 21) and GPx (CAT. No. GP 2524) were determined according to (Paglia and Valentine 1967) and (Nishikimi et al. 1972), respectively.

### Immune response attributes

The levels of blood serum interleukin-6 (IL-6), interferons (IFN-γ), and tumor necrosis factors (TNF-α) were measured via immune-enzymatic assays using commercially-available ELISA. Bovine IL-6 was determined using the calorimetric method (My BioSource, San Diego, USA Cat No. MBS733925), with a sensitivity of up to 1.0 pg/mL. IFN-γ was determined using a specialized quantitative competitive method using an ELISA kit (My BioSource, San Diego, USA, Cat No. MBS70468), with a detection range of 8–200 pg/ml and a sensitivity of up to 5 pg/ml, whereas the intra- and inter-assay precision was ≤ 8% and 10% respectively. TNF-α was determined using a specialized quantitative sandwich ELISA kit (My BioSource, San Diego, USA, Cat No. MBS2609886) with a detection range of 15.6–1000 pg/ml and a sensitivity of up to 5 pg/ml, whereas the intra- and inter-assay precision was ≤ 8% and ≤ 12%, respectively. Immunoglobulin G (IgG) and lysozyme activity were also measured. IGg levels were detected using an ELISA technique using commercial kits and following the manufacturer’s instructions (Artursson et al. 1995; Wattrang et al. 1997). The stander range of the assay was 15.625–1000 ng/ml and no cross reactivity was recorded at 50 ng/ml for other species (human, mouse, and rat). A lysoplate assay was used to assess lysozyme activity, as described by Lie et al. (1986) using Micrococcus lysodeikticus mixed in agarose 1% as a substrate.

### Statistical analysis

A multivariate mixed effects logistic regression model (PROC LOGISTIC; SAS Institute Inc. 2012) was run with the level of significance set at α = 0.05 to examine the effects of potential risk factors, including BCS, gestation period, calf sex, dam parity, calving season, and calving year, on the occurrence of SB. The statistical model was incorporated as follows:

$$\text{log}\left(\left(\pi STB\right)/\left(1-\pi STB\right)\right)=\beta 0+\beta 1F+\beta 2BCS+\beta 3GP+\beta 4S+\beta 5P+\beta 6SE+ \beta 7Y$$

where πSTB is the probability of SB occurrence, $\upbeta 0\text{ is the intercept},$ and $\beta 1F, \beta 2BCS,\beta 3GP, \beta 4S, \beta 4S, \beta 5P, \beta SE, {\mathrm{and}}\ \beta 7Y$ are regression for farm effect, BCS, gestation period (GP), calf sex (S), dam parity (P), calving season (SE), and calving year (CY).

In the statistical model, BCS, GP, S, P, SE, and CY were included as fixed effects, while the farm was considered a random effect. The CY was included in the model only to control for its effects on dependent variables. Confidence intervals (95% CI) were described according to Schwabe (1982). Significant differences between explanatory variables were tested using a Chi-squared test (χ^2^). SB rates were calculated as the number of calves that died shortly after delivery divided by the total number of calves born. Survival rates were calculated as the number of live calves after the critical period of SB occurrence divided by the total number of calves born. Data were edited in Microsoft Excel (Microsoft Corporation, Redmond, WA, USA). Differences in blood parameters between SB- and live-born calves were detected using a student’s t-test in Microsoft Excel. The sample size was detected according to the Thompson equation at CI = 95%, Z = 1.96, α = 0.05, D = 0.05, and P = 0.50 (Thompson 2012). Statistical significance was set at *p-*value less than 0.05.

## Results

### Overall distribution of SB, and survival rates

**Fig. 1:**
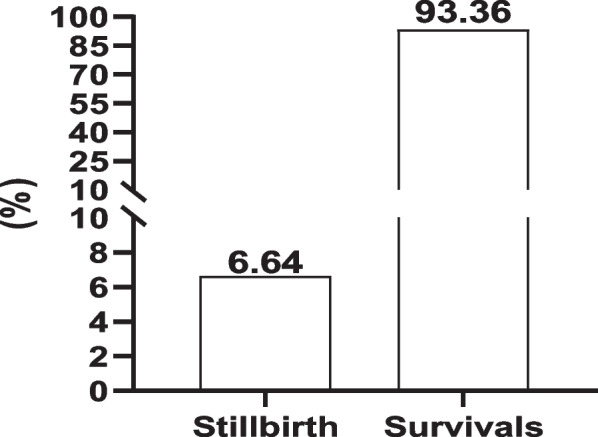
The distribution of stillbirth cases (%) in Egyptian buffaloes

The prevalence of SB and survival rates is illustrated in Fig. 1. The percentage of SB was 6.64% and the survival rate percentage was 93.36%.

## Risk factors for SB

The potential risk factors associated with the probability of SB occurrence in Egyptian buffaloes are shown in Tables 1 and 2. BCS, GP, parity number, calving season, sex, and calving year had significant effects (*p* < 0.05) on the occurrence of SB occurrence. The odds of SB were greater in obese animals (OR = 1.877; 87.7%) and lower in both average (OR = 0.386; 61.4%) and fat (OR = 0.191; 80.9%) animals, compared to thin animals. Animals with a GP ≥ 305 days were 73.7% (OR = 0.246) less likely to produce SB calves than those with a G*P* < 305 days. Interestingly, SB risk decreased steadily with increasing parity number; the probability of giving SB calves decreased by 12.7% (OR = 0.873) for buffaloes in their second parity and increased by 67.4% (OR = 0.326) for those in their fifth parity. In comparison with the spring season, the probability of SB occurrence increased by two times (OR = 2.015) during the summer season. Moreover, male calves had 2.476 times greater odds of SB than female calves. Regression analysis indicated that the probability of SB occurrence decreased by 58.9% (OR = 0.411) by calving year (2020–2023).

**Table 1:** The distribution of stillbirth cases (%) in Egyptian buffaloes according to various risk factors, including body condition score, gestation period, calf sex, dam parity, and calving season

Item^1^	*n*	%	Χ^2^	*p-*value
Body condition score			10.73	0.01
Thin (2 – 2.5)	31	37.15		
Average (3 – 3.5)	20	24.10		
Fat (4 – 4.5)	10	12.05		
Obese (5)	22	26.50		
Gestation period			14.8	0.0001
< 305 days	24	28.92		
≥ 305 days	59	71.08		
Sex			24.4	< 0.0001
Female	19	22.89		
Male	64	77.11		
Parity number			27.06	< 0.0001
1st	29	34.94		
2^nd^	26	31.33		
3rd	15	18.07		
4th	7	8.43		
5th	6	7.23		
Season			41.77	< 0.0001
Spring	14	16.87		
Summer	44	53.01		
Autumn	21	25.30		
Winter	4	4.82

**Table 2:** Logistic regression analysis of different risk factors associated with the probability of the occurrence of stillbirth (SB) in Egyptian buffaloes

Item	*n*	β	OR	95% CI	*p-*value
Body condition score					
Thin (2 – 2.5)	277		Ref		
Average (3 – 3.5)	431	− 0.95	0.386	0.215–0.692	0.002
Fat (4 – 4.5)	426	− 1.66	0.191	0.091–0.295	> 0.001
Obese (5)	115	0.63	1.877	1.196 – 1.501	0.038
Gestation period					
< 305 days	137		Ref		
≥ 305 days	1112	− 1.33	0.264	0.159–0.440	> 0.001
Sex					
Female	513		Ref		
Male	736	0.91	2.476	1.464–4.186	> 0.001
Parity number					
1st	292		Ref		
2nd	296	− 0.14	0.873	0.630–1.409	0.633
3rd	326	− 0.83	0.437	0.251–0.620	0.012
4th	162	− 0.89	0.410	0.245–0.583	0.039
5th	173	− 1.12	0.326	0.138–0.562	0.015
Season					
Spring	331		Ref		
Summer	460	0.87	2.015	1.242–3.767	0.006
Autumn	357	0.35	1.415	0.906–1.931	0.326
Winter	101	− 0.07	0.933	0.530–1.411	0.906
Calving year (2013 – 2020)					
Linear Trend	1249	− 1.26	0.411	0.269–0.719	> 0.001

*β* regression coefficient, *OR* odds ratio, *CI* confidence interval (95%), *Ref*. reference

### Biochemical blood parameters

The levels of the biochemical parameters measured in the SB and control groups are shown in Table 3. The levels of serum uric acid, creatinine, AST, ALT, LHD, GGT, cholesterol, bilirubin, amylase, and lipase were significantly (*p* < 0.05) higher in the SB group than in normal-born calves. There were no significant differences in the levels of serum urea and total protein between SB-born and normal-born calves.

**Table 3:** Levels of blood serum biochemistry in stillbirth (SB) and normal calves (mean ± SE)

Item^1^	SB-born calves (*n* = 10)	Normal-born calves (*n* = 10)	*p-*value
Total protein (g/dl)	6.8 ± 0.25	6.7 ± 0.23	0.861
Cholesterol(mg/dl)	78.0 ± 1.88	54.0 ± 1.34	0.011
Liver function indicators			
AST (IU/l)	150.0 ± 3.63	109.0 ± 2.96	0.013
ALT(IU/l)	53.0 ± 2.85	18.0 ± 1.29	0.009
GGT (Ul)	5.43 ± 0.86	1.5 ± 0.04	0.001
LDH (mg/dl)	644.0 ± 8.63	420.0 ± 6.81	0.001
Bilirubin (mg/dl)	3.6 ± 0.34	0.63 ± 0.16	0.001
Kidney function indicators			
Uric Acid (mg/dl)	1.7 ± 0.13	0.78 ± 0.23	0.001
Creatinine (mg/dl)	0.97 ± 0.03	0.67 ± 0.01	0.037
Pancreas function indicators			
Amylase(mg/dl)	19.0 ± 0.71	12.0 ± 0.56	0.001
Lipase (mg/dl)	68.0 ± 1.11	60.0 ± 0.98	0.003

^1^*AST* aspartate aminotransferase, *ALT* alanine aminotransferase, *GGT* gamma-glutamyl transferase, *LDH* lactate dehydrogenase

### Antioxidant indicators

Table 4 shows a significant increase (*p* < 0.05) in the levels of serum MDA and a significant decrease in the GPx activity in SB-born calves, in comparison with normal-born calves. There were no significant differences in the levels of serum TAC and SOD between SB- and normal-born calves.

**Table 4:** Levels of serum redox status of stillbirth (SB) and normal calves (mean ± SE)

Item^1^	SE-born calves (*n* = 10)	Normal-born calves (*n* = 10)	*p-*value
TAC(ng/ml)	0.685 ± 0.02	0.713 ± 0.04	0.136
SOD(U/ml)	69.14 ± 5.36	82.21 ± 7.03	0.086
GPx(U/ml)	84.23 ± 3.91	150.37 ± 6.15	0.001
MDA(nmol/ml)	5.06 ± 1.04	0.351 ± 0.01	0.001

^1^*TAC* total antioxidant capacity, *SOD* superoxide dismutase, *GPx* glutathione peroxidase, *MDA* malondialdehyde

### Immune parameters

The levels of proinflammatory cytokines (IL6, IFN-γ, and TNF-α), major immunoglobulin classes (IgG), and lysosome activity (LYZ) in both SB- and normal-born calves are shown in Table 5. The levels of IL-6, IFN-γ, and TNF-α were higher (p = 0.001) in the SB group than in the control group. In contrast, the levels of IgG and LZY activity were lower (*p* = 0.001) in SB-born calves than in normal-born calves.

**Table 5:** Concentrations of serum immune responses and proinflammatory cytokinesis in stillbirth (SB) and normal calves (mean ± SE)

Item^1^	Stillbirth Cases	Normal Cases	*p-*Value
IL-6 (pg/ml)	340.36 ± 4.17	237.17 ± 3.92	0.001
IFN-γ (pg/ml)	601.28 ± 5.62	350.41 ± 4.41	0.001
TNF-α (pg/ml)	136.20 ± 3.55	58.50 ± 2.69	0.001
LYZ(ng/ml)	0.595 ± 0.03	3.09 ± 0.21	0.001
IgG(ng/ml)	68.32 ± 2.63	84.22 ± 2.34	0.001

^1^*IL-6* interleukin-6, *IFN* interferons gamma, *TNF-α* tumor necrosis factors, *LYZ* lysosome activity, *IgG* immunoglobulins G

## Discussion

Stillbirth (SB) has been identified as one of the major reproductive disorders in Egyptian buffaloes (Salem and Amin 2017). In the present study, the overall SB rate was 6.6%. A similar trend was observed by Nasr (2017),who found that the overall SB rates for primiparous and multiparous Egyptian × Italian buffalo crosses were 12.4% (*n* = 1965) and 9.2% (*n* = 6.423), respectively. In fact, the observed SB rates for Egyptian buffaloes in our study and the Egyptian × Italian crosses in the study by Nasr (2017) can be considered threatening rates, as most buffalo farms in Egypt encompass small- and medium-sized herds (Abdel-Salam and Fahim 2018). Thus, when reproductive losses due to SB alone reach such rates, significant reproductive wastage is expected when such source of reproductive failure gather to other common reproductive disorders such as failing to conceive after breeding such abortion, retained placenta, and purulent vaginal emancipation (Deka et al. 2021). These findings highlight the importance of identifying the different potential risk factors for this type of reproductive loss.

In this study, we analyzed SB risk factors and determined the metabolic status, redox status, and immune-inflammatory biomarkers in a small sample of both SB- and live-born calves. These biomarkers may reflect the response of animals to environmental conditions, internal maternal circumstances, and fetal wellbeing during the late stages of pregnancy and parturition. Previous studies concluded that BCSat calving has a substantial influence on the prevalence of SB in cows (Chassagne et al. 1999; Berry et al. 2007); the current results demonstrated a similar relationship. This relationship with BCScan be mainly ascribed to the fact that obese/over-conditioned animals have extreme lipids in the pelvic region, which reduces the pelvic area, reducing perinatal welfare and potentially increasing the prevalence of SB through dystocia (Meijering 1984; Mellado et al. 2017). However, dystocia was not recorded in the present study in stillbirth calves.

The outcomes of this screening discovered that the odds of SB were higher in the summer months, which is in settlement with the results of previous investigations (Hossein-Zadeh et al. 2008; Al-Samarai 2012; El-Tarabany 2015). However, in this study, the summer season represented a major risk factor, rather than other seasons. Other studies noted that spring (Johanson and Berger 2003; Atashi 2011) or winter (Uematsu et al. 2013) were SB-related seasons, mainly due to a longer GP with heavy calf birth weights (Fourichon et al. 2001), and decreased survival rates at lower temperatures (Azzam et al. 1993). With respect to our findings, under tropical and subtropical conditions, high climate temperatures along with elevated humidity during the summer season can negatively affect maternal health and welfare, as well as alter physiological responses (Essawi et al. 2021). It is also important to consider the increased SB rates in autumn and spring. In Egypt, the main source of roughage is Trifolium alexandrinum (Egyptian clover), which contains considerable concentrations of phytoestrogens, which disrupt endocrine system, during pregnancy, mainly by disrupting progesterone concentrations ( Hashem et al. 2016). These findings suggest that phytoestrogens may interfere with the hormonal balance of pregnant buffaloes, increasing pregnancy complications and thus increasing the rates of SB, however, no direct evidence confirms this assumption. Therefore, more studies are required to study the relationship between phytoestrogens and the occurrence of SB.

The odds of SB calves were lower in buffaloes with a GP ≥ 305 days, compared to those with a G*P* < 305 days; these results correspond with the verdicts of Mellado et al. (2017), who noted that lactating Holstein cows with a gestation length < 278 days were 9 times (OR = 9; CL = 7.9–10.2) more likely to produce SB calves compared to those with a gestation length > 278 days. Moreover, dam parity had a significant effect on SB. From first parity onwards, the incidence of SB decreased. These upshots are in agreement with the consequences obtained from a previous study (Nasr 2020). The inverse relationship between dam parity and SB rate may be related to incongruities in calf weight, health and the BCS of cow, in addition to vulvar and pelvic conformation. An increase in parity corresponds to increasing occurrences of calving ease in buffaloes due to a larger pelvic area (Ghav et al. 2012; El-Regalaty 2014; Amin et al. 2021). The increased SB rates observed in first parity buffaloes and those who delivered at a shorter gestation length may be related to intrauterine fetal growth restriction during pregnancy and/or the incomplete development of some physiological systems, such as the immune system (SB-born calves had lower IgG levels); this may be a result of prioritizing energy expenditure, directing energy to maternal tissues to meet both the maintenance and growth requirements of dams ( Grimard et al. 2006; Cabezas-Garcia et al. 2021).

There has been conflicting evidence for the impact of calf sex on the incidence of SB (Amin et al. 2021); however, generally, there is an increased rate of SB in male calves (Ghav et al. 2012). The present findings confirm that male calves have poorer vitality at birth than female calves. Male sex steroids (testosterone and other androgens) are known to have an immuno-suppressive effect, whereas female sex steroids (estrogen) have an immuno-enhancing effect on the immune system. The effect of calf sex could feasibly be owing to the greater weight of male calves, causing an extended parturition time and the need for delivery assistance. Unpleasant conditions around and during parturition may cause severe acidosis as a significance of oxygen absence and low blood pH, with resultant impacts on the performance of calf energetic structures (viz. brain, spleen and hepatic) and overall survivability and vitality (Smith 2000; Ghav et al. 2012; Ahmadpanah et al. 2023).

In this study, the metabolic status, redox status, and immune-inflammatory biomarkers were determined in SB- and live-born calves. In fact, these biomarkers may reflect the response of animals to environmental conditions, internal maternal circumstances, and fetal wellbeing during the late stages of pregnancy and around parturition. It is important to note that the selected SB-born calves were born to healthy dams, and they did not suffer from dystocia at birth. Thus, these calves died due to idiopathic etiology rather than infection and/or dystocia. According to the results of the blood serum analysis, SB-born calves demonstrated higher immune-inflammatory responses (higher concentrations of cytokines and IgG), weaker redox defense systems (lower concentrations of antioxidant enzymes), and impaired functioning of some vital organs, including the liver, kidney, and pancreas, compared to live-born calves. During pregnancy, the fetus can develop a local or systemic immune-inflammatory reaction when an organism exposed to infection-related stimuli, showing danger signals or alarmins (Jung et al. 2020). Although the systemic fetal inflammatory reaction is assumed to be an imperative adaptive physiological event for survival, it may become dysfunctionality, thereby a fetal cytokine rainstorm results and can result to manifold organ dysfunction/impairment, such as brain damage, bronchopulmonary dysplasia, and fetal short-term perinatal mortality and morbidity (Jung et al. 2020). Higher levels of cytokines, such as IL-6, IL-1B, and TNF-α, in dams and/or fetal circulatory cycles are related to increased cases of pre-term delivery (Gotsch et al. 2007).

Many factors may lead to impaired immune-inflammatory responses and redox defense system during fetal life; the most common is related to infectious diseases. However, in our study, all dams and/or calves were in good health, suggesting the presence of other factors leading to these responses. According to the literature, in dairy animals, one of the major drivers of the occurrence of inflammation and other immune responses is a negative energy balance (LeBlanc 2012; Chastant and Saint-Dizier 2019; Hashem et al. 2021). From three weeks before and three weeks postpartum (the transition period), dairy animals faces a negative energy balance and oxidative stress, together with digestive acidosis and social stress, all situations that can put dams in a proinflammatory situation and lead to immune dysfunction, increasing susceptibility to reproductive tract inflammatory and/or non-infection-related disease (Chastant and Saint-Dizier 2019). Moreover, in the last trimester of pregnancy, many immune-inflammatory responses are developed to contribute to the initiation of the parturition process. Fetal major histocompatibility complex Class 1 molecules begin to be expressed by placental cells, initiating a maternal response. Leukocytes are recruited through the placenta via several chemoattracting cytokines (TNF-α, IL-8 and IL-2) and phagocyte placental cells. The activities of matrix metallo proteinase and collagenase enzymes increase in the maternal and fetal parts of the placenta (Chastant and Saint-Dizier 2019); however, these changes, which are required to initiate parturition and placenta expulsion, if increased and accompanied with other inflammatory-driven factors, can worsen placenta competence and lead to the loosening and subsequently the detachment of villi (Chastant and Saint-Dizier 2019; Moradi et al. 2022). In an in vitro study by Hill and Gilbert (2008), induced non-infectious endometrial inflammation decreased the number of trophectoderm; this may lead to decreases in placental weight from day 42 of pregnancy, if pregnancy is maintained (Lucy et al. 2016).

Based on the results of the risk analysis performed in this study, we infer that some risk factors may induce a severe disruption in the calf’s metabolism and immune responses, increasing the hazards of SB. For example, SB-born calves demonstrated disrupted liver and kidney functions and a lower ability to neutralize reactive oxygen species, as indicated by lower antioxidant enzyme activities and higher MDA levels compared to normal-born calves. In fact, these disruptions could be easily related to the effect of heat stress. High ambient temperature impairs the redox status of animals, increases the release of reactive oxygen species, and decreases placenta competence (Monteiro et al. 2016). Moreover, utero heat stress during late pregnancy has long-term effects on the fetal programming of postnatal growth and disease susceptibility (Monteiro et al. 2016). The increased levels of creatinine, urea, and uric acid in the serum of SB-born calves could be due to the effect of high temperature on cellular lysis from tissues such as skeletal muscle and red blood cells and/or the accumulation of this metabolites due to liver and/or kidney dysfunction (Donaldson and Lamont 2015). These effects may explain the higher rate of SB cases during the summer season.

In this study, obese buffaloes expressed higher SB rates than average-sized and thin buffaloes. Obesity triggers many inflammatory reaction cascades (Yan et al. 2011), which were also seen in the SB-born calves in this study. These outcomes are in track with those obtained from studies of the obese sheep model, in which maternal obesity evokes inflammation and enhances the transcript of proinflammatory cytokines (transforming growth factor beta and IL-7) in the large intestine of fetuses and offspring (Yan et al. 2011).

According to the results of the present study, we conclude that factors related to the induction of inflammation and/or disrupted immune system responses and the redox status of calves, such as obesity and high temperature, are the main risk factors for SB in buffaloes. Additionally, the insufficient body maturation of dams appears to play a crucial role in increasing the number of SB cases. Based on these findings, several measures or interventions can be applied to minimize the risk of SB in buffaloes: 1- adjusting the BCS of dams around parturition and ensuring sufficient maturity of nulliparous and primiparous dams; 2- maintaining a suitable ambient temperature; 3- monitoring the type of feed and its secondary metabolites; and 4- controlling the calves’ sex, if possible.

## Conclusion

These results represent the first attempt to describe the risk factors associated with the incidence of SB in newborn Egyptian buffaloes. The putative risk factors associated with SB were BCS, GP, calf sex, dam parity, and calving season. The cause of SB in calves that appeared to be clinically normal and born at parturition with no apparent cause for mortality is likely to be multifactorial and the screening of immunoglobulins, antioxidant, immune markers, and the metabolic profile in buffalo calves may offer valuable contributions to further clarifying the cause of SB in buffalo herds. Moreover, we conclude that factors related to the induction of inflammation and/or disrupted immune system responses, such as dam obesity, high environmental temperature, and oxidative stress, are the main evoking factors for SB in buffaloes; therefore, protective measures against SB in Egyptian buffaloes should be based on controlling these factors, either by nutritional interventions or management practices. 

## Authors^,^ contributions

Conceptualization: AAEl-R, WME, MAEH and SAA; methodology: AA El-R, WME, MAEH and SAA; investigation: AA El-R, WME, MAEH and SAA; resources: AA El-R, WME, MAEH and SAA; data curation: AAEl-R, WME, MAEH, SAA and NMH; Writing original draft preparation: AAEl-R, SAA and NMH; review and editing: AAEl-R, and NMH. All authors have read and agreed to the manuscript's current published version.

## Data Availability

The data presented in this study are available on request from the corresponding author.
